# Russian Adaptation of Questionnaire of Mental Health Treatment Stigma among Adolescents: Preliminary Results

**DOI:** 10.1192/j.eurpsy.2022.1117

**Published:** 2022-09-01

**Authors:** A. Khromov

**Affiliations:** MENTAL HEALTH RESEARCH CENTER (1); Moscow State University of Psychology & Education (2), Department Of Medical Psychology (1); Department Of Neuro And Pathopsychology (2), Moscow, Russian Federation

**Keywords:** Questionnaire, self-stigma, Adolescents, Mental Health treatment

## Abstract

**Introduction:**

There is a lack of instruments evaluating self-stigma among adolescents with mental health issues in the Russian language for today. The questionnaire developed by Tally Moses (Moses, 2009) is convenient to fill that lack.

**Objectives:**

The study aims to compare the main parameters of the original questionnaire to that of the version translated in Russian.

**Methods:**

The original questionnaire was translated into Russian and administered to 40 adolescents (21 males, aged 12 to 17) with mental disorders except for severe cognitive deficits or pervasive developmental disorders. Means and Cronbach’s alpha for each of the four scales were assessed and compared to the author’s questionnaire values.

**Results:**

Reliability analysis revealed similar Cronbach’s alpha for 3 of 4 scales (table 1) except the Secrecy scale (1 of 6 questions showed low consistency; its exclusion increased α from 0.63 to 0.74).

**Table:** 

***Scales***	***Cronbach’s α***		***M (SD)***		
	***Original version***	***Translated version***	***Original version***	***Translated version***	***t-test***
Societal Devaluation	.76	.76	2.3 (0.40)	2.3 (0.42)	.501
Personal Rejection	.78	.70	0.48 (0.39)	0.33 (0.29)	*.002*
Self-Stigma	.81	.76	2.0 (0.74)	2.2 (0.68)	.122
Secrecy scale	.84	.63	2.5 (0.50)	2.5 (0.55)	.594

The means for each scale were compared with original data using a one-sample t-test. Only the Personal Rejection scale was significantly low on average than the original data.

**Conclusions:**

Preliminary results showed that Russian adolescent patients perceived the translated questionnaire much the same way as American ones. Thus, our findings provide optimistical perspectives of further adaptation of the questionnaire.

**Disclosure:**

No significant relationships.

